# Author Correction: Increased autophagy in EphrinB2-deficient osteocytes is associated with elevated secondary mineralization and brittle bone

**DOI:** 10.1038/s41467-019-13040-5

**Published:** 2019-11-04

**Authors:** Christina Vrahnas, Martha Blank, Toby A. Dite, Liliana Tatarczuch, Niloufar Ansari, Blessing Crimeen-Irwin, Huynh Nguyen, Mark R. Forwood, Yifang Hu, Mika Ikegame, Keith R. Bambery, Cyril Petibois, Eleanor J. Mackie, Mark J. Tobin, Gordon K. Smyth, Jonathan S. Oakhill, T. John Martin, Natalie A. Sims

**Affiliations:** 1 Bone Biology and Disease Unit, St. Vincent’s Institute of Medical Research, 9 Princes Street, Fitzroy, Melbourne, VIC 3065 Australia; 2 Department of Medicine, The University of Melbourne, St. Vincent’s Hospital, Melbourne, VIC 3065 Australia; 3 Metabolic Signalling Laboratory, St. Vincent’s Institute of Medical Research, 9 Princes Street, Fitzroy, Melbourne, VIC 3065 Australia; 4 Department of Veterinary Biosciences, Melbourne Veterinary School, The University of Melbourne, Parkville, VIC 3010 Australia; 5 School of Medical Science and Menzies Health Institute Queensland, Griffith University, Gold Coast, QLD 4222 Australia; 6 Bioinformatics Division, The Walter and Eliza Hall Institute of Medical Research, Parkville, VIC 3010 Australia; 7 Department of Oral Morphology, Graduate School of Medicine, Dentistry and Pharmaceutical Sciences, Okayama University, Okayama, 700-8525 Japan; 8 Infrared Microspectroscopy (IRM) Beamline, ANSTO Australian Synchrotron, Clayton, VIC 3168 Australia; 9 University of Bordeaux, Inserm U1029 LAMC, Allée Geoffroy Saint-Hilaire Bat. B2, 33600 Pessac, France; 10 School of Mathematics and Statistics, The University of Melbourne, Melbourne, VIC 3010 Australia; 11 Mary MacKillop Institute for Health Research, Australian Catholic University, Melbourne, VIC 3065 Australia; 12 Present Address: MRC Protein Phosphorylation and Ubiquitylation Unit, James Black Centre, University of Dundee, Dundee, DD1 4HN UK

**Keywords:** Bone development, Bone remodelling, Autophagy

Correction to: *Nature Communications* 10.1038/s41467-019-11373-9, published online 31 July 2019.

The original version of this Article contained an error in Figure 3. In panel b, the legend embedded in the panel was incorrect and indicated a solid grey line for w/w and a dotted black line for f/f. The correct legend is now provided and shows a dotted black line for w/w and a solid grey line for f/f. This has been corrected in both the PDF and HTML versions of the Article.

