# Supplementary material for: Increased autophagy in EphrinB2-deficient osteocytes is associated with elevated secondary mineralization and brittle bone
Source: Nat Commun. 2019 Jul 31;10:3436. doi: 10.1038/s41467-019-11373-9 (PMC6668467; doi:10.1038/s41467-019-11373-9)
Supplement: Supplementary file 1 — Supplementary Information [file 41467_2019_11373_MOESM1_ESM.pdf]

Supplementary Information

Increased autophagy in EphrinB2-deficient osteocytes is associated with elevated secondary mineralization and brittle bone

Vrahnas et al

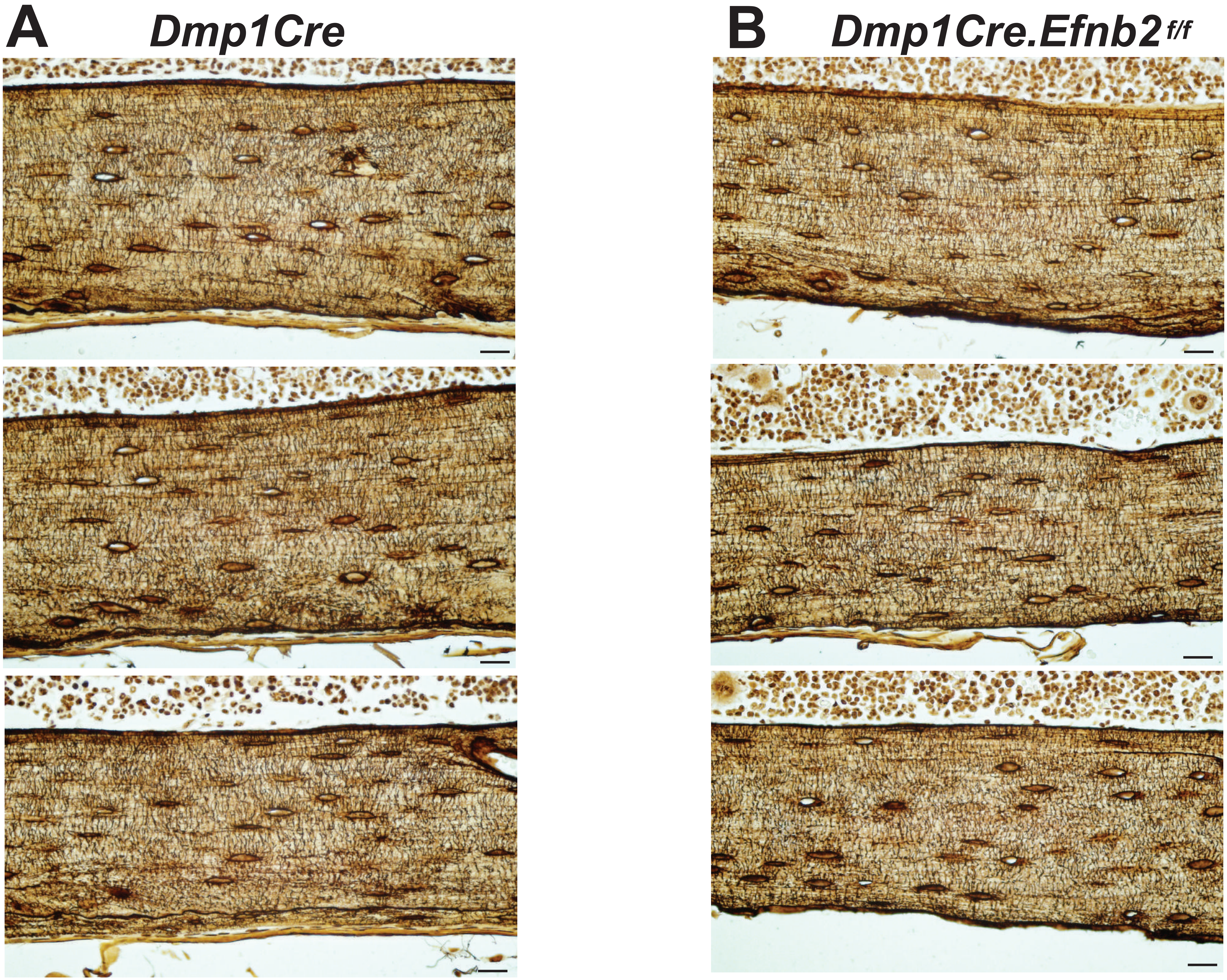

Supplementary Figure 1: No obvious change in the osteocyte lacunar-canalicular network with EphrinB2 deletion. Comparison is shown between (A) *Dmp1Cre* (w/w) and (B) *Dmp1Cre.Efnb2*$^{f/f}$ (f/f) tibial cortex; images of Ploton silver stain taken at the medial diaphysis from 3 littermate pairs at 12 weeks of age. Scale bar = 20 micron.

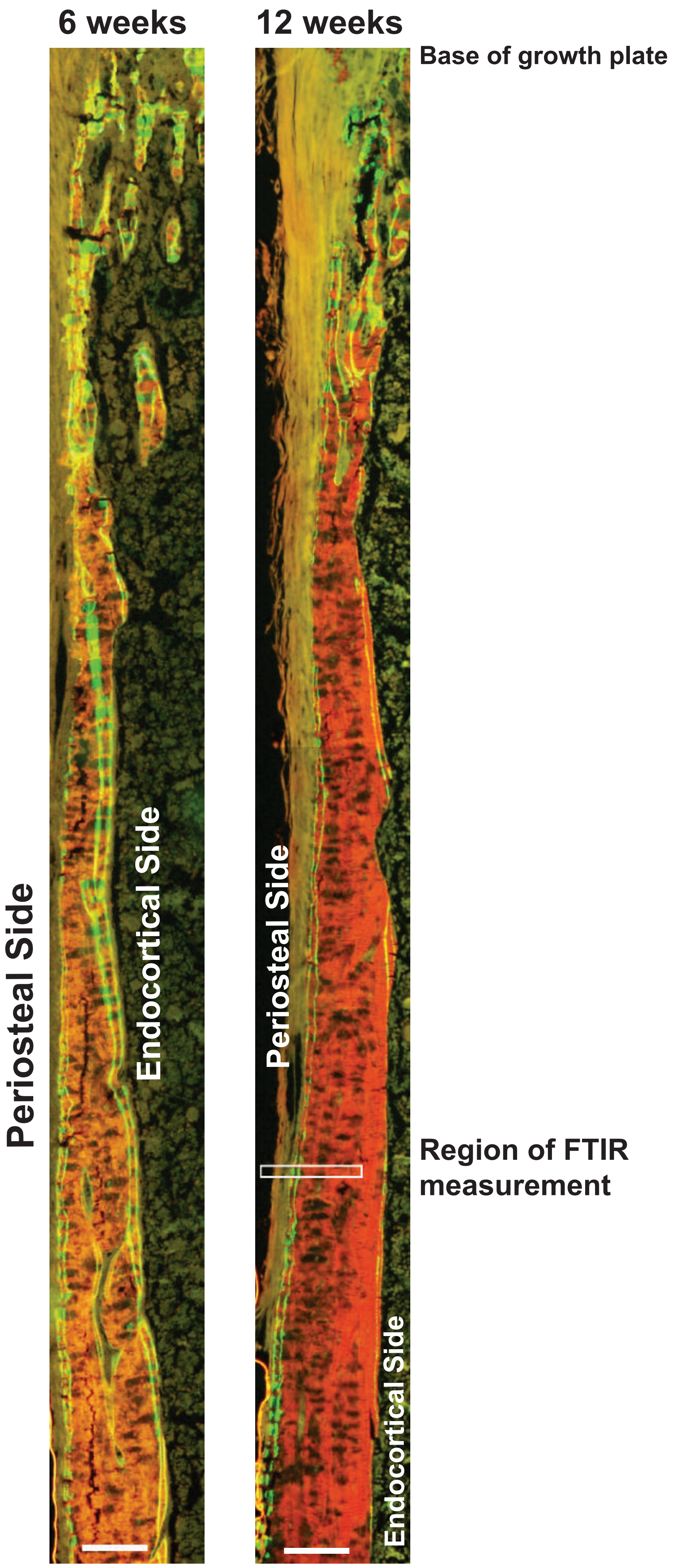

Supplementary Figure 2: Xylenol orange-stained sections of 6 and 12 week old *Dmp1Cre* tibial cortical bone showing double calcein labels (green) administered at 3 and 10 days prior to tissue collection. Note the continuous double label on the periosteal bone surface (left hand side) at both ages that extends from the base of the growth plate to the region measured by sFTIRM (white box) in the 12 week old specimen. The endocortical bone surface also shows double label, but it is not continuous at 12 weeks, indicating regions that are either inactive, or have undergone resorption. Scale bar = 100 micron.

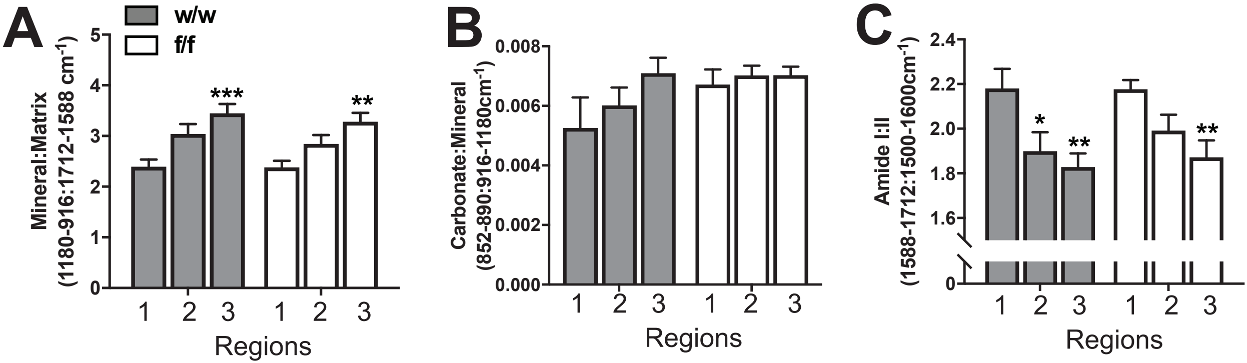

Supplementary Figure 3: No change in mineral accrual, carbonate deposition, or amide I:II ratio in male *Dmp1Cre.Efnb2*$^{f/f}$ (f/f) tibial cortical bone. sFTIRM-derived mineral:matrix (A), carbonate:mineral (B), and amide I:II (C) in regions 1-3 of *Dmp1Cre* (w/w) and f/f littermate tibiae; regions defined in Figure 4. Data are represented as mean ± SEM, n = 10/group. *p<0.05, **p<0.01, ***p<0.001 vs region 1 of same genotype (bone maturation effect). No significant effect of genotype was detected.

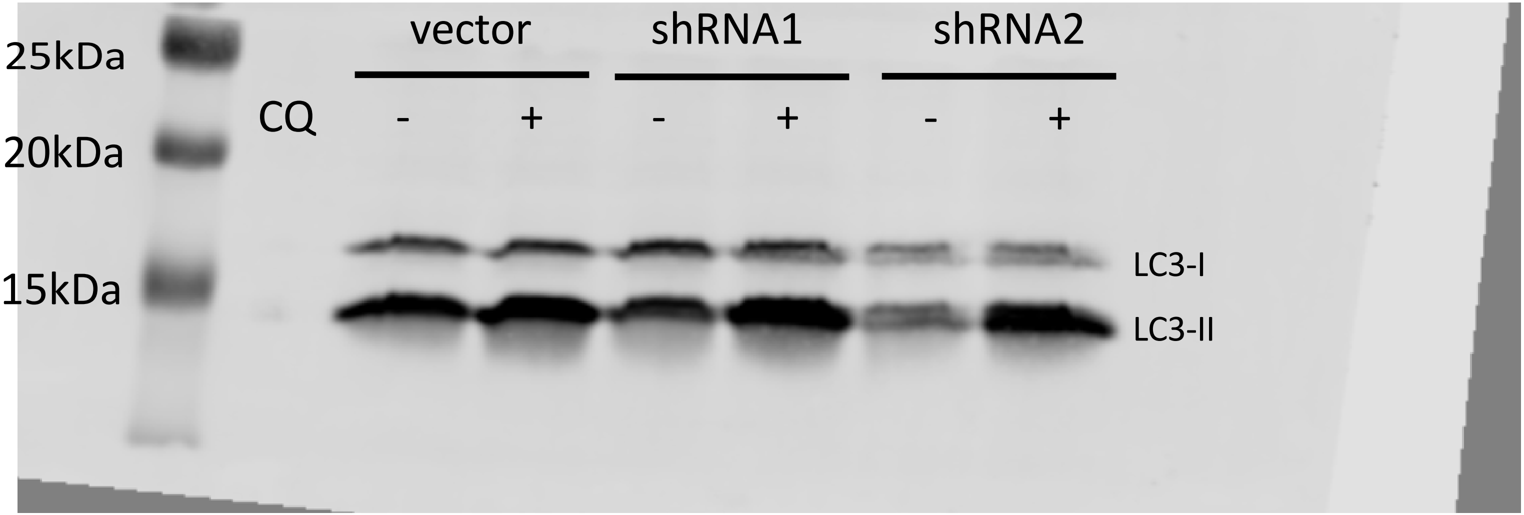

Supplementary Figure 4: Uncropped and unprocessed scan of Western Blot from Figure 5d.

Supplementary Information

Supplementary Tables

Supplementary Table 1. Additional structural and material properties and histomorphometry of *Dmp1Cre.Efnb2$^{f/f}$* femoral cortical bone

	Dmp1Cre	*Dmp1Cre.Efnb2$^{f/f}$*
Yield Force (N)	14.98 ± 0.63	11.15 ± 0.90**
Yield deformation (mm)	0.18 ± 0.01	0.12 ± 0.01**
Stiffness (N/mm)	139.32 ± 10.16	134.59 ± 10.31
Anteroposterior (AP) width (mm)	1.24 ± 0.02	1.25 ± 0.02
Mediolateral (ML) width (mm)	1.80 ± 0.03	1.75 ± 0.01
Yield Stress (MPa)	31.96 ± 2.12	24.15 ± 1.81*
Yield Strain (%)	0.04 ± 0.002	0.02 ± 0.001**
Elastic Modulus (MPa)	1430.5 ± 105.8	1320.4 ± 93.5
Periosteal Perimeter (μm)	6.90 ± 0.13	6.86 ± 0.07
Marrow Area (mm^2)	0.94 ± 0.04	0.97 ± 0.02
Periosteal Mineral Appositional Rate (μm/day)	0.98 ± 0.12	1.07 ± 0.10
Periosteal Mineralizing Surface / Bone Surface (%)	88.41 ± 7.45	80.82 ± 6.30
Cortical Tissue Mineral Density (g/cm^3)	1.13 ± 0.02	1.13 ± 0.02

Data are from 12 week old female mice, shown as mean ± SEM; n = 10-12/group. $^{*}p < 0.05$, $^{**}p < 0.01$, versus *Dmp1Cre* controls, Student's t-test.

Supplementary Table 2. 3-point bending tests results for 12-week old male *Dmp1Cre.Efnb2$^{f/f}$* femora and *Dmp1Cre* controls.

	Dmp1Cre	*Dmp1Cre.Efnb2$^{f/f}$*
Ultimate Force (N)	22.03 ± 1.18	23.85 ± 1.43
Ultimate Deformation (mm)	0.29 ± 0.03	0.28 ± 0.03
Post-Yield Deformation (mm)	0.21 ± 0.40	0.17 ± 0.29
Energy absorbed to failure (mJ)	5.25 ± 0.77	5.00 ± 0.66
Moment of inertia (mm^4)	0.99 ± 0.10	0.97 ± 0.07
Ultimate Stress (MPa)	25.31 ± 2.18	26.98 ± 1.85
Ultimate Strain (%)	0.07 ± 0.01	0.06 ± 0.01
Toughness (mJ/mm^3)	1.48 ± 0.26	1.35 ± 0.19

Data are represented as mean ± SEM; n = 12/group

Supplementary Table 3. Reference point indentation parameters measured on the cortical mid-shaft of femora from 12-week old female and male *Dmp1Cre.Efnb2$^{f/f}$* mice, compared to *Dmp1Cre* controls.

	Female		Male	
	Dmp1Cre	*Dmp1Cre.Efnb2$^{f/f}$*	*Dmp1Cre*	*Dmp1Cre.Efnb2$^{f/f}$*
Indentation Distance – 1st cycle (μm)	19.43 ± 0.44	19.75 ± 0.36	19.45 ± 0.33	20.16 ± 0.55
Total Indentation Distance (μm)	20.77 ± 0.75	21.18 ± 0.38	20.63 ± 0.34	21.53 ± 0.57
Average Loading Slope (N/μm)	0.31 ± 0.01	0.29 ± 0.01	0.31 ± 0.01	0.29 ± 0.01
Unloading slope – 1st cycle (N/μm)	0.39 ± 0.02	0.37 ± 0.02	0.38 ± 0.02	0.35 ± 0.02
Average Unloading Slope (N/μm)	0.41 ± 0.02	0.38 ± 0.02	0.40 ± 0.02	0.37 ± 0.02
Creep Indentation Distance – 1st cycle (μm)	1.57 ± 0.09	1.65 ± 0.10	1.60 ± 0.12	1.65 ± 0.09
Average Creep Indentation Distance (μm)	0.58 ± 0.10	0.62 ± 0.11	0.57 ± 0.08	0.65 ± 0.10
Average Energy Dissipated (μJ)	2.47 ± 0.16	2.60 ± 0.18	2.32 ± 0.18	2.28 ± 0.12

Data are represented as mean ± SEM; n = 11-12/group

Supplementary Table 4. MicroCT analysis of trabecular bone structure in 12-week old female and male *Dmp1Cre.Efnb2$^{f/f}$* mice, compared to controls.

	Female		Male	
	Dmp1Cre	*Dmp1Cre.Efnb2$^{f/f}$*	*Dmp1Cre*	*Dmp1Cre.Efnb2$^{f/f}$*
Trabecular Bone Volume (%)	8.87 ± 0.68	10.38 ± 0.71	16.49 ± 0.96^{+}	16.02 ± 0.92
Trabecular Thickness (μm)	53.98 ± 0.64	53.66 ± 0.70	60.35 ± 0.82^{+}	60.34 ± 0.73
Trabecular number (/mm)	1.64 ± 0.12	1.93 ± 0.12	2.73 ± 0.15^{+}	2.65 ± 0.14
Trabecular Separation (μm)	340.17 ± 22.24	266.60 ± 15.22**	221.49 ± 15.58^{+}	225.61 ± 17.71

Data are represented as mean ± SEM; n = 12/group

**p<0.01 vs. sex-matched *Dmp1Cre.Efnb2 $^{w/w}$* controls, Two-way Anova, Fisher's LSD test

$^{+}$ p<0.001 vs. female *Dmp1Cre.Efnb2 $^{w/w}$* controls, Two-way Anova, Fisher's LSD test

Supplementary Table 5. Histomorphometric analyses of female trabecular bone

	Dmp1Cre	*Dmp1Cre.Efnb2* $^{f/f}$
Mineral Apposition Rate (µm/day)	2.09 ± 0.09	1.96 ± 0.10
Mineralizing surface/Bone Surface (%)	34.51 ± 2.52	34.87 ± 1.04
Bone Formation Rate/Bone Surface (µm^2/µm/day)	0.71 ± 0.04	0.68 ± 0.04
Osteoid Thickness (µm)	2.26 ± 0.17	2.66 ± 0.21
Osteoid Surface (%)	7.39 ± 1.45	9.83 ± 2.29
Osteoblast Surface (%)	13.60 ± 1.81	16.25 ± 2.20
Osteoclast Surface (%)	8.78 ± 1.25	11.58 ± 0.95

12-week old female *Dmp1Cre.Efnb2* $^{f/f}$ tibiae compared to sex and age-matched *Dmp1Cre* controls. Data are represented as mean ± SEM; n = 7-11/group.

Supplementary Table 6. Collagen fiber deposition and osteocyte lacunar parameters.

	Dmp1Cre	*Dmp1Cre.Efnb2*$^{f/f}$
Woven bone (% total bone)	17.00 ± 1.58	19.33 ± 2.30
Average osteocyte lacunar size (µm^2)	35.27 ± 1.09	35.76 ± 0.83
Osteocyte lacunar size - largest 20% (µm^2)	59.81 ± 2.05	60.11 ± 2.05
Osteocyte lacunar density (/mm^2)	232.91 ± 12.27	268.75 ± 9.26[a]
Whole bone hydroxyproline (µg/mg protein)	36.38 ± 3.37	44.51 ± 2.87[a]

Data from the femoral midshaft of 12-week old female *Dmp1Cre* (w/w) and *Dmp1Cre.Efnb2*$^{f/f}$ (f/f) mice. Data are represented as mean ± SEM; n = 10-12/group $^{*}p < 0.05$ versus *Dmp1Cre* controls, Student's t-test.

Supplementary Table 7. Primers used for qRT-PCR:

Gene name – protein name	Forward (5'-3')	Reverse (5'-3')	Reference
Efnb2 - EphrinB2-	AGAACTGGGAGCGGCTTG	TGGCCAACAGTTTTAGAGTCC	1
B2m - beta-2-microglobulin	TTCACCCCCACTGAGACTGAT	GTCTTGGGCTCGGCCATA	2
Hmbs - hydroxymethylbilane synthase	TCATGTCCGGTAACGGCG	CACTCGAATCACCCTCATCTTTG	3
Hprt1 - hypoxanthine phosphoribosyltransferase 1	TGATTAGCGATGATGAACCAG	AGAGGGCCACAATGTGATG	4

References to Supplementary Table 7:

1. Tonna S, *et al.* EphrinB2 signaling in osteoblasts promotes bone mineralization by preventing apoptosis. *FASEB J* **28**, 4482-4496 (2014).

2. Winkler IG, Hendy J, Coughlin P, Horvath A, Lévesque J-P. Serine protease inhibitors serpina1 and serpina3 are down-regulated in bone marrow during hematopoietic progenitor mobilization. *The Journal of Experimental Medicine* **201**, 1077-1088 (2005).

3. Walsh NC, *et al.* Osteoblast function is compromised at sites of focal bone erosion in inflammatory arthritis. *J Bone Miner Res* **24**, 1572-1585 (2009).

4. Onan D, *et al.* The chemokine Cxcl1 is a novel target gene of parathyroid hormone (PTH)/PTH-related protein in committed osteoblasts. *Endocrinology* **150**, 2244-2253 (2009).
