# Supplementary material for: Increased autophagy in EphrinB2-deficient osteocytes is associated with elevated secondary mineralization and brittle bone
Source: Nat Commun. 2019 Jul 31;10:3436. doi: 10.1038/s41467-019-11373-9 (PMC6668467; doi:10.1038/s41467-019-11373-9)
Supplement: Supplementary file 3 — Reporting Summary [file 41467_2019_11373_MOESM3_ESM.pdf]

nature research

Corresponding author(s): Natalie A Sims

Last updated by author(s): Jul 3, 2019

Reporting Summary

Nature Research wishes to improve the reproducibility of the work that we publish. This form provides structure for consistency and transparency in reporting. For further information on Nature Research policies, see Authors & Referees and the Editorial Policy Checklist.

Statistics

For all statistical analyses, confirm that the following items are present in the figure legend, table legend, main text, or Methods section.

n/a	Confirmed	
☐	☒	The exact sample size (*n*) for each experimental group/condition, given as a discrete number and unit of measurement
☐	☒	A statement on whether measurements were taken from distinct samples or whether the same sample was measured repeatedly
☐	☒	The statistical test(s) used AND whether they are one- or two-sided<br>*Only common tests should be described solely by name; describe more complex techniques in the Methods section.*
☒	☐	A description of all covariates tested
☐	☒	A description of any assumptions or corrections, such as tests of normality and adjustment for multiple comparisons
☒	☐	A full description of the statistical parameters including central tendency (e.g. means) or other basic estimates (e.g. regression coefficient) AND variation (e.g. standard deviation) or associated estimates of uncertainty (e.g. confidence intervals)
☒	☐	For null hypothesis testing, the test statistic (e.g. *F*, *t*, *r*) with confidence intervals, effect sizes, degrees of freedom and *P* value noted<br>*Give P values as exact values whenever suitable.*
☒	☐	For Bayesian analysis, information on the choice of priors and Markov chain Monte Carlo settings
☒	☐	For hierarchical and complex designs, identification of the appropriate level for tests and full reporting of outcomes
☒	☐	Estimates of effect sizes (e.g. Cohen's *d*, Pearson's *r*), indicating how they were calculated

Our web collection on statistics for biologists contains articles on many of the points above.

Software and code

Policy information about availability of computer code

Data collection	*Provide a description of all commercial, open source and custom code used to collect the data in this study, specifying the version used OR state that no software was used.*
Data analysis	Histomorphometry was performed using Osteomeasure software; BSEM analysis used MetaMorph v7.8.3.0; micro-computed tomography used NRecon (version 1.6.9.8), Dataviewer (version 1.4.4) and CT Analyser (version 1.11.8.0); sFTIRM data acquisition was undertaken with Bruker OPUS version 6.5 and data analysis used OPUS version 7.2; FTIRI used CytoSpec 1.4.0.3; Bioinformatics used Rsubread, featureCounts and limma software. Confocal microscopy was quantified using ImageJ (version 1.48v) and MetaMorph (version 7.8.6.0)

For manuscripts utilizing custom algorithms or software that are central to the research but not yet described in published literature, software must be made available to editors/reviewers. We strongly encourage code deposition in a community repository (e.g. GitHub). See the Nature Research guidelines for submitting code & software for further information.

Data

Policy information about availability of data

All manuscripts must include a data availability statement. This statement should provide the following information, where applicable:

- Accession codes, unique identifiers, or web links for publicly available datasets
- A list of figures that have associated raw data
- A description of any restrictions on data availability

RNA sequencing data generated for this study is deposited with GEO, accession number GSE110795. All other data is available from the corresponding author on request.

Field-specific reporting

Please select the one below that is the best fit for your research. If you are not sure, read the appropriate sections before making your selection.

☒ Life sciences ☐ Behavioural & social sciences ☐ Ecological, evolutionary & environmental sciences

For a reference copy of the document with all sections, see nature.com/documents/nr-reporting-summary-flat.pdf

Life sciences study design

All studies must disclose on these points even when the disclosure is negative.

Sample size	Sample sizes were chosen based on previously published works using similar methods.
Data exclusions	No data was excluded.
Replication	Replication is reported in all figure legends. Replication of in vivo studies was achieved by using a large sample size; Replication of in vitro studies was achieved by repeating each experiment independently on 3 separate occasions with triplicate wells in each experiment.
Randomization	No treatments were carried out on mice, so randomization was not a part of this study.
Blinding	Investigators were blinded to animal genotype and sex or cell treatment group for all analyses conducted. This includes micro-computed tomography, histomorphometry, confocal microscopy, and PCR analysis.

Reporting for specific materials, systems and methods

We require information from authors about some types of materials, experimental systems and methods used in many studies. Here, indicate whether each material, system or method listed is relevant to your study. If you are not sure if a list item applies to your research, read the appropriate section before selecting a response.

Materials & experimental systems

n/a	Involved in the study	
☐	☒	Antibodies
☐	☒	Eukaryotic cell lines
☒	☐	Palaeontology
☐	☒	Animals and other organisms
☒	☐	Human research participants
☒	☐	Clinical data

Methods

n/a	Involved in the study	
☒	☐	ChIP-seq
☒	☐	Flow cytometry
☒	☐	MRI-based neuroimaging

Antibodies

Antibodies used	Immunofluorescence: LC3 primary antibody (MBL, M152-3); Goat anti-mouse (Invitrogen AlexaFluor 488 goat anti-mouse cross-adsorbed secondary antibody A21121). Western blot: LC3B (D11, Cell Signaling Technology, 3868), goat anti-rabbit IgG antibody tagged with IR680 (LI-COR Biosciences).
Validation	LC3 primary antibody: Validated by the manufacturer for immunoflurescence and in 25 citations; goat anti-mouse AlexaFluor 488 validated by the manufacturer for immunofluorescence and used in 29 citations; LC3B primary for Western blotting was validated by the manufacturer for Western Blotting; and the goat antirabbit igG was validated by the manufacturer (LI-COR) for Western blot imaging using the Odyssey system.

Eukaryotic cell lines

Policy information about cell lines

Cell line source(s)	Ocy454 cells were obtained from Dr Paola Divieti-Pajevic; Kusa 4b10 cells were subcloned by our laboratory.
Authentication	None of the cells lines are commercially available and were not authenticated.
Mycoplasma contamination	All cell lines tested negative for mycoplasma contamination.
Commonly misidentified lines (See ICLAC register)	N/A.

Animals and other organisms

Policy information about studies involving animals; ARRIVE guidelines recommended for reporting animal research

Laboratory animals	Mus musculus, Tg(Dmp1-Cre)<1Jqfe>.Efnb2<tm1And>, backcrossed onto C57BL/6 for 6 generations. All experiments use mice that are sex and age- matched littermates.
Wild animals	The study did not involve wild animals
Field-collected samples	The study did not involve samples collected from the field.
Ethics oversight	Ethical approval was obtained from the Animal Ethics Committee of St. Vincent's Health Australia (Melbourne)

Note that full information on the approval of the study protocol must also be provided in the manuscript.
